# The function of integron-associated gene cassettes in *Vibrio* species: the tip of the iceberg

**DOI:** 10.3389/fmicb.2013.00385

**Published:** 2013-12-09

**Authors:** Rita A. Rapa, Maurizio Labbate

**Affiliations:** ^1^ithree Institute, University of TechnologySydney, NSW, Australia; ^2^Department of Medical and Molecular Biosciences, University of TechnologySydney, NSW, Australia

**Keywords:** integron, gene cassette, *Vibrio*, mobile DNA, mobile genetic elements, mobile genes, lateral gene transfer

## Abstract

The integron is a genetic element that incorporates mobile genes termed gene cassettes into a reserved genetic site via site-specific recombination. It is best known for its role in antibiotic resistance with one type of integron, the class 1 integron, a major player in the dissemination of antibiotic resistance genes across Gram negative pathogens and commensals. However, integrons are ancient structures with over 100 classes (including class 1) present in bacteria from the broader environment. While, the class 1 integron is only one example of an integron being mobilized into the clinical environment, it is by far the most successful. Unlike clinical class 1 integrons which are largely found on plasmids, other integron classes are found on the chromosomes of bacteria and carry diverse gene cassettes indicating a non-antibiotic resistance role(s). However, there is very limited knowledge on what these alternative roles are. This is particularly relevant to *Vibrio* species where gene cassettes make up approximately 1–3% of their entire genome. In this review, we discuss how emphasis on class 1 integron research has resulted in a limited understanding by the wider research community on the role of integrons in the broader environment. This has the capacity to be counterproductive in solving or improving the antibiotic resistance problem into the future. Furthermore, there is still a significant lack of knowledge on how gene cassettes in *Vibrio* species drive adaptation and evolution. From research in *Vibrio rotiferianus* DAT722, new insight into how gene cassettes affect cellular physiology offers new alternative roles for the gene cassette resource. At least a subset of gene cassettes are involved in host surface polysaccharide modification suggesting that gene cassettes may be important in processes such as bacteriophage resistance, adhesion/biofilm formation, protection from grazers and bacterial aggregation.

## INTRODUCTION

Members of the *Vibrio* genus are ubiquitous in marine environments and show a wide range of niche specialization (Thompson et al., 2004). The capability of vibrios to occupy diverse niches is a testament to their ability to adapt and evolve. An important driver of this in vibrios is lateral gene transfer (LGT). LGT is the mechanism of DNA transfer from one bacterial cell to another without the requirement for cell division. It is followed by subsequent incorporation of the DNA into the recipients’ genome such that DNA can be stably inherited, a process assisted by mechanisms such as homologous recombination or via a range of mobile genetic elements (MGEs) such as transposons and genomic islands (Stokes and Gillings, 2011). This mini review will focus on one important MGE called the integron, an element commonly known for its role in antibiotic resistance. The focus on the integron and its role in antibiotic resistance has driven a lack of understanding (and perhaps lack of interest) for the role this element plays in the broader environment. In contrast, we argue that understanding integron contribution to the antibiotic resistance problem requires an understanding of the role of integrons in their broad evolutionary context. Since integrons are present in almost all *Vibrio* species and comprise a significant proportion of their genome, they are excellent candidates for studying alternative roles of integrons outside of the clinical environment. Using recent work from *Vibrio rotiferianus* DAT722, we discuss possible environmental roles for this MGE.

## WHAT ARE INTEGRONS?

**FIGURE 1:**
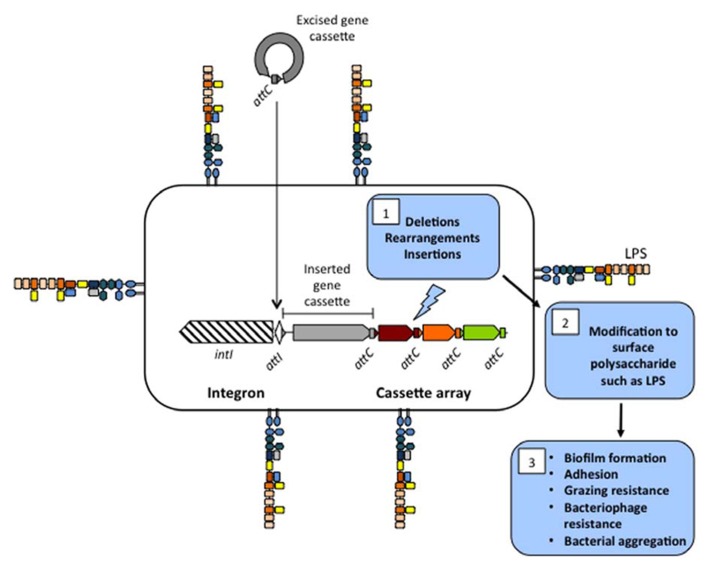
**Proposed mechanism for the production of surface polysaccharide diversity through deletions, rearrangements, and insertions in the cassette array of *Vibrio* species.** The integron consists of an integrase gene (*intI*) and a primary recombination site called *attI*. Gene cassettes consists of a secondary recombination site called *attC* and are circularized in their excised format. Recombination of multiple cassettes into *attI* (or *attC*) results in a contiguous cassette array that in *Vibrio* species can make up 1–3% of their genome. Deletion, rearrangements, and insertions in the cassette array (1) results in modification of cell surface polysaccharide (2) that may affect processes such as biofilm formation, adhesion to different surfaces, grazing resistance, bacteriophage resistance, and bacterial aggregation (3).

An integron is a site-specific recombination system capable of integrating and expressing open reading frames (ORFs) contained in modular structures called gene cassettes (**Figure 1**; Mazel, 2006; Labbate et al., 2009). The integron is defined by three components, an integrase gene (*intI*) that encodes a site-specific recombinase, an attachment site (*attI*), and a promoter (P_c_). The mobile units that insert into integrons are gene cassettes. Gene cassettes commonly consist of a single promoterless ORF and an IntI-identifiable recombination site called *attC.* The integration of gene cassettes is facilitated by an integrase-mediated recombination reaction between *attI* × *attC* and less commonly *attC* × *attC*. Multiple insertion events produce a contiguous cassette array with cassettes downstream of the P_c_ promoter being co-transcribed. Induction of *intI* can also cause excision and rearrangement of a gene cassette(s) into a different position.

Integrons are a diverse family of elements and are catalogued into classes based on the nucleotide sequence of the integrase gene. Currently, there are over 100 different integron classes, *most present on bacterial chromosomes* and found in approximately 10% of sequenced bacterial genomes (Boucher et al., 2007). The class 1 integron was the first described integron (Stokes and Hall, 1989), found linked to antibiotic resistance genes in resistance plasmids from Gram negative pathogens. This is because class 1 integrons in clinical contexts largely contain antibiotic resistance gene cassettes with approximately 130 so far described (Partridge et al., 2008). The accumulation of multiple resistance gene cassettes (up to about six) has associated these elements with multi-drug resistance (Leverstein-van Hall et al., 2002, 2003). Unlike clinical class 1 integrons which carry an identical integrase gene sequence, diverse class 1 integrons are also found in the chromosomes of environmental *Betaproteobacteria,* containing divergent integrase sequences and functionally diverse gene cassettes. This indicates that *Betaproteobacteria* were the original source of the clinical class 1 integron and that its initial capture by a transposon disseminated it across diverse Gram negative pathogens and human commensals (Gillings et al., 2008).

Although the class 1 integron is by far the most abundant integron in clinical contexts, others have been described (approximately five). An environmental source for all these clinical integrons strongly suggests that integrons have a much broader role in adaptation than conferring resistance to antibiotics in clinical environments (Rowe-Magnus et al., 2001; Boucher et al., 2007; Gillings et al., 2008). Phylogeny shows integrons to be ancient structures (Rowe-Magnus et al., 2001; Boucher et al., 2007) therefore, the gene cassette pool has been contributing to adaptation and evolution of bacteria for several hundred million years and not just in the last 70 years during the antibiotic revolution. This point is sometimes not well understood by researchers studying clinically derived integrons.

## RESEARCHING THE BROADER ROLE OF INTEGRONS IN A FIELD FOCUSED ON THEIR CONTRIBUTION TO THE DISSEMINATION OF ANTIBIOTIC RESISTANCE

Due to the ongoing issue of bacterial antibiotic resistance, research is still heavily focused on the role of class 1 integrons. A PubMed search with the term “integron” retrieves in excess of 2200 publications. A search with the following terms “integron and (antibiotic or resistance or class 1)” retrieves 1847 publications showing that 83% focus on antibiotic resistance and/or class 1 integrons. Antibiotic resistance is a significant issue and we are certainly not suggesting that the emphasis on the role of integrons in this area is not justified however, we believe that this is impacting adversely on our understanding of these elements including in relation to the antibiotic resistance problem. Firstly, the focus on antibiotic resistance is skewing understanding for the general role of integrons in the wider research community. Given the hundreds of integron classes that exist, much of our knowledge is based on a single class (i.e., the class 1 integron). This overshadows the likely important role that integrons play in the broader environment and sometimes results in an erroneous dogma that knowledge of class 1 integrons can be extrapolated to all integron classes. The overshadowing of research in integrons outside the clinical setting is easily observed via a cursory examination of the literature over the last 3 years. Although we have known for nearly a decade that there are over 100 classes of integrons (most from non-clinical bacteria including in *Vibrio* species), publications still claim the existence of “4” (Madiyarov et al., 2010; Rezaee et al., 2012), “5” (Li et al., 2013), “10” (Salimizand et al., 2013), or “several” (Peymani et al., 2012) classes of integrons based on their knowledge of clinically derived integrons.

We and other authors have also experienced erroneous dogma in comments provided by expert reviewers for submitted manuscripts, particularly with regard to whether integrons described from natural environments correspond with what is known about “integrons” (mostly class 1). An excellent example as to why research from class 1 integrons cannot be extrapolated to all integron classes is shown in the recombination reaction rate of the class 1 integron and that from *Vibrio cholerae*. The *V. cholerae* integron has a 2600-fold higher rate of recombination in a *V. cholerae* background compared to an *Escherichia coli* background indicating the involvement of host factors (Biskri et al., 2005). In contrast, the class 1 integron shows no difference in rates of recombination in both backgrounds. The class 1 integron’s capacity to operate in different backgrounds is the likely reason for why this particular integron has been successful in its mobilization across different bacteria. This trait and possibly a greater capacity to integrate cassettes with diverse *attC* sites (Biskri et al., 2005) are likely to make this integron an exception rather the rule.

Secondly, it has been over 12 years since the discovery that integrons are diverse and found in different environments (Mazel et al., 1998; Nield et al., 2001). Knowledge on the function of integrons with regard to site-specific recombination, transcription of gene cassettes, and regulation of the integron-integrase has significantly advanced and has been excellently reviewed elsewhere (Cambray et al., 2010; Roy Chowdhury et al., 2011). However, little progress has been made in addressing the precise ways in which gene cassette products contribute to the adaptation and evolution of bacteria outside of antibiotic resistance. Based on our knowledge of integrons in antibiotic resistance, we are aware of the power of this system in providing rapid adaptation under strong selection pressure(s). In approximately 70 years, the integron has assisted in making antibiotic treatment problematic and most likely obsolete in the next 10 years (World Health Organisation, 2013). Class 1 integrons are now a common fixture on plasmids from commensal bacteria and Gram negative pathogens. Re-entry of commensal and Gram negative pathogens into the broader environment through routes such as wastewater treatment ensures that access to the environmental gene cassette metagenome will be easy and rapid. Thus, a lack of understanding or distribution of misinformation regarding this greater resource, particularly in the antibiotic/clinical field, has the potential to be counterproductive in the quest to solve or improve the antibiotic resistance problem into the future.

In addressing the knowledge gap for the broader adaptive role of integrons, *Vibrio* species make excellent candidates. As already stated the cassette arrays of vibrios tend to be quite large comprising up to 3% of their genome and consisting of diverse and unique gene cassettes. To date, the largest cassette array is in *Vibrio vulnificus* CMCP6 consisting of 219 cassettes and totaling approximately 150 kb (Kim et al., 2003). In order to highlight the necessity for research into the role integrons play in bacterial adaptation and evolution and to focus attention on the lack of understanding that exists about the function of this element in bacteria generally, we will review and discuss the phenotypic functions of cassettes in *Vibrio* species in the context of what has been discussed above.

## A BIG BLACK BOX IN OUR UNDERSTANDING OF GENE CASSETTE FUNCTION

Cassette arrays in *Vibrio* species are large and mostly consist of unique and novel genes with no identifiable function. In 2007, a bioinformatics survey of gene cassettes from multiple genome sequenced *Vibrio* species found that 65% of cassette proteins had no known homologs and that 13% had homologs of unknown function (Boucher et al., 2007). The remainder showed a wide range of non-specific functions in metabolism, cellular processes, and information storage. Similar statistics have been observed through PCR amplification of gene cassettes from metagenomic DNA (Elsaied et al., 2007; Koenig et al., 2008, 2009). Putting aside this massive knowledge gap in cassette function for the moment, large cassettes arrays provide an extra level of complexity. While some have argued that P_c_ is the only driver of cassette transcription in large arrays (Guerin et al., 2009; Cambray et al., 2010), other studies have shown otherwise (Yildiz et al., 2004; Michael and Labbate, 2010). A study of the 116-gene cassette array of *V. rotiferianus* DAT722 showed that the majority of gene cassettes were transcribed and that numerous diverse promoters across the array were present that responded to different growth conditions (Michael and Labbate, 2010). The presence of these diverse promoters provides integrated cassettes with multiple regulatory options. This gives the capacity for cassettes to re-arrange with different promoters potentially building operon-like structures that express complimentary cassette proteins. Such an idea has been demonstrated in principal using artificial gene cassettes containing genes for tryptophan biosynthesis (Bikard et al., 2010). This complexity can be elevated when we consider that *Vibrio* species live in populations where gene cassettes might be considered a community resource not just a singular cell resource. For example, integrons might provide a way for the community to break down and/or extract energy from complex substrates without the entire pathway (and genetic burden) being owned by just one cell. Amusingly, the complexity of integrons has been used as proof for God/intelligent design (Hunter, 2010).

Even with the limited understanding of gene cassette function, a number of studies have sampled the gene cassette metagenome from different environments and attempted to determine how cassettes might influence adaptation and evolution (Elsaied et al., 2007, 2011, 2013; Koenig et al., 2008, 2009, 2011). Although correlations are observed such as homologs of genes in cassettes encoding pollution degrading enzymes from contaminated environments (Nemergut et al., 2004; Koenig et al., 2009) or environments showing a “gene cassette ecotype” (Koenig et al., 2008), it is still the case that ~80% of the gene cassettes are of unknown function. In a study looking at gene cassettes from *Vibrio* species found in coral mucus, 12.5% of gene cassettes were implicated in biochemical processes also associated with antibiotic resistance (Koenig et al., 2011). The authors argued that gene cassettes provide a competitive advantage by delivering protection from, or by synthesizing, antimicrobials in the coral environment. While sound, the fact that this conclusion could be drawn clearly reflects the amount of research that has been done in the integron/resistance field. No other conclusions on the other cassette-assisted bacterial interactions present in the coral mucus could be made. So we are still left with a gaping hole in our understanding of how gene cassettes contribute to adaptation and/or evolution in this environment.

A handful of gene cassette products have been functionally characterized and these have been summarized in **Table 1**. In many instances, characterization of these gene cassettes was selected based on some homology to a known protein such that a phenotype could be tested which does not really address the bulk of unknown and hypothetical gene cassette products. In other instances, they were selected based on their capacity to be crystallized or were identified as part of mutant library or other screens. In the instances where gene cassettes have been removed from their natural bacterial host and expressed in *E. coli* or where *in vitro* techniques have been used to study protein activity, caution must be taken in how their function is interpreted. Interactions of cassette proteins with host pathways may modify how these gene cassettes affect cell or community behavior. This was observed in a study in *V. rotiferianus* DAT722 where deletion of a gene cassette encoding a putative topoisomerase I-like protein affected porin regulation. This phenotype could not have been predicted if characterized outside the host (Labbate et al., 2011). This is also true of the bioinformatic studies described above where in the small proportion of gene cassette products that could be identified were often proteins such acetyltransferases, methylases, or transcriptional regulators. Without knowing the primary substrate that is being modified by the acetyltransferase or methylase or the gene(s) controlled by the transcriptional regulator, the biological importance of the cassette(s) is still unclear. Therefore, an approach where gene cassettes are deleted or expressed in their natural host is arguably the best way to identify their physiological role.

**Table 1:** Non-antibiotic resistance and experimentally confirmed functional ORFs in gene cassettes.

Source of cassette	Function	Determination of function	Reference
**Cassettes from *Vibrio* species**
*Vibrio cholerae*	Sulfate-binding protein	Complementation of *E. coli* mutation	Rowe-Magnus et al. (2001)
*Vibrio cholerae* OP4G	Transcriptional regulation	Crystal structure determination and drug binding assay	Deshpande et al. (2011)
*Vibrio cholerae* GP156	Heat stable enterotoxin	Active in suckling mouse assay when expressed in *E. coli*	Ogawa and Takeda (1993)
*Vibrio cholerae*	Mannose–fucose resistant hemagglutinin	Mutagenesis *in vivo* and testing in infant mouse model	Franzon et al. (1993), Barker et al. (1994)
*Vibrio marinus*	Psychrophilic lipase	Active when expressed in *E. coli* at 10^°^C	Rowe-Magnus et al. (2001)
*Vibrio vulnificus* CMCP6**	Cold shock	Complementation of cold shock phenotype in *E. coli*	Rowe-Magnus (2009)
*Vibrio vulnificus* CMCP98K	Secretion	Expression in *E. coli* mediates secretion of periplasmic proteins	Kim et al. (2003)
*Vibrio rotiferianus* DAT722 (cassette 21)**	dNTP-pyrophosphohydrolase (iMazG)	Crystal structure determination. Expressed in *E. coli* and enzyme activity measured	Robinson et al. (2007)
Various large cassette arrays like this in *Vibrio* spp.	Toxin/antitoxin (TA) genes	Demonstration that presence of TA genes limits deletions in large cassette arrays	Szekeres et al. (2007)
*Vibrio vulnificus* 1003	Capsular polysaccharide biosynthesis	Transposon mutagenesis *in vivo*	Smith and Siebeling (2003)
*Vibrio rotiferianus* DAT722 (cassette 11)**	Porin regulation	Deletion of cassette *in vivo*	Labbate et al. (2011)
*Vibrio rotiferianus* DAT722 (multiple cassettes)**	Surface polysaccharide modification	Deletion of cassettes *in vivo*	Rapa et al., 2013
**Cassettes from metagenomic DNA**
Soil metagenomic DNA	Potential transport protein	Crystal structure determination	Robinson et al. (2005)
Soil metagenomic DNA	ATPase activity	Expressed in *E. coli* and enzyme activity measured	Nield et al. (2004)
Soil metagenomic DNA	Methyltransferase activity	Expressed in *E. coli* and enzyme activity measured	Nield et al. (2004)

## NEW INSIGHT INTO GENE CASSETTE FUNCTION IN THE VIBRIOS

*Vibrio rotiferianus* DAT722 is the only microorganism where extensive physiological analysis has been conducted on isogenic mutants with gene cassettes deleted. This has revealed new insights into how gene cassettes affect adaptation and evolution. In one study, a cassette could not be deleted without a compensatory mutation (Labbate et al., 2011). The resulting mutants had abnormal regulation of their porins and impaired growth in minimal medium. The gene cassette in question was the 11th cassette from *attI* and appears to be strain specific by lacking close relatives elsewhere. The cassette 11 protein contains two domains, one with weak homology to nucleases and the other a C4-zinc finger domain commonly found in topoisomerase 1 proteins. These domains indicate a DNA binding/processing protein that is likely to have a regulatory role potentially through controlling the coiling state of gene promoters. Irrespective of the exact role, this study is important in demonstrating that cellular networks can rapidly integrate a mobile gene cassette such that it becomes advantageous for survival. It also shows that benefit need not necessarily come from acquisition of a novel functional gene(s) but through modification of existing host cellular networks (Labbate et al., 2012).

In a follow up to this study, the impact of deletions on the cassette array of *V. rotiferianus* DAT722 was addressed (Rapa et al., 2013). Indels are regularly observed in *V. cholerae* arrays and are likely in all large arrays however, their impact on bacterial physiology were unknown (Labbate et al., 2007; Szekeres et al., 2007; Yan et al., 2011). Three deletion mutants were subjected to physiological growth, stress, proteomic, and chemistry-based techniques to determine the effect of cassette deletions on vibrio physiology. The total deleted cassettes encompassed 58% of the array. Surprisingly, growth and stress assays of these mutants showed little change compared to the wild-type. Furthermore, proteomic analysis of one deletion mutant in different media and growth stages showed only 0.5–1% change in the proteome. This indicates that unlike deletion of cassette 11, the majority of cassettes are not integrated into host pathways and do not affect the major metabolic pathways of the cell, at least in the conditions observed.

Importantly, analyses did identify changes to host surface polysaccharide in the deletion mutants with proton nuclear magnetic resonance on whole cell polysaccharide indicating that gene cassette products decorate host cell polysaccharide via the addition or removal of functional groups. Consistent with this result, one mutant had modified biofilm-forming capabilities in a simple batch biofilm assay (Rapa et al., 2013). This is a significant result as it focuses future researchers who are addressing gene cassette function in vibrios to surface polysaccharide. We propose that at least a subset of cassettes are involved in modifying host surface polysaccharide and that deletion (and most likely rearrangements and acquisition) of cassettes is a mechanism for creating surface property diversity. There is significant biological importance to surface-associated polysaccharide and its modification as evidenced in the literature. This includes biofilm formation (Lee et al., 2013), bacterial cell co-aggregation (Vu et al., 2009), bacteriophage resistance (Scholl et al., 2005), evasion of immune cells (Pier et al., 2001) as well as resistance to antimicrobial peptides (Westman et al., 2008; **Figure 1**).

## CONCLUSION

In the last 12 years, little progress has been made in the precise ways that cassette gene products contribute to adaptation and evolution of *Vibrio* species. One reason is the emphasis that is placed on studying integrons from clinical contexts. Another is that characterization of unknown genes is difficult and thus not considered a fruitful endeavor by researchers, especially in the current competitive research environment. However, if we are to learn more about the broader role of integrons, some of our focus needs to shift to identifying functions for gene cassettes. This will not only improve our understanding of this important genetic resource in a broader sense but give improved context for these elements clinically.

## Conflict of Interest Statement

The authors declare that the research was conducted in the absence of any commercial or financial relationships that could be construed as a potential conflict of interest.

## AUTHOR CONTRIBUTIONS

Rita A. Rapa and Maurizio Labbate both contributed to the writing of this manuscript.
